# Saikosaponins induced hepatotoxicity in mice *via* lipid metabolism dysregulation and oxidative stress: a proteomic study

**DOI:** 10.1186/s12906-017-1733-0

**Published:** 2017-04-19

**Authors:** Xiaoyu Li, Xiaojiaoyang Li, Junxian Lu, Youyi Huang, Lili Lv, Yongfu Luan, Runping Liu, Rong Sun

**Affiliations:** 1 Department of Medical Pathomorphology, Shandong Academy of Traditional Chinese Medicine, Jinan, 250014 Shandong China; 2 Jiangsu Key Laboratory of Drug Screening, China Pharmaceutical University, Nanjing, Jiangsu 210009 China; 3 Department of Microbiology and Immunology, Virginia Commonwealth University, Richmond, VA 23220 USA; 4 The Post of Taishan Scholar in Traditional Chinese Medicine Pharmacology and Toxicology Expert, Jinan, Shandong 250014 China; 5 Third Degree Laboratory of Chinese Medicine Pharmacology, State Administration of Traditional Chinese Medicine, Shandong Academy of Traditional Chinese Medicine, Jinan, 250014 Shandong China

## Abstract

**Background:**

Radix Bupleuri (RB) has been popularly used for treating many liver diseases such as chronic hepatic inflammation and viral Hepatitis in China. Increasing clinical and experimental evidence indicates the potential hepatotoxicity of RB or prescriptions containing RB. Recently, Saikosaponins (SS) have been identified as major bioactive compounds isolated from RB, which may be also responsible for RB-induced liver injury.

**Methods:**

Serum AST, ALT and LDH levels were determined to evaluate SS-induced liver injury in mice. Serum and liver total triglyceride and cholesterol were used to indicate lipid metabolism homeostasis. Liver ROS, GSH, MDA and iNOS were used to examine the oxidative stress level after SS administration. Western blot was used to detect CYP2E1 expression. A 8-Plex iTRAQ Labeling Coupled with 2D LC - MS/MS technique was applied to analyze the protein expression profiles in livers of mice administered with different doses of SS for different time periods. Gene ontology analysis, cluster and enrichment analysis were employed to elucidate potential mechanism involved. HepG2 cells were used to identify our findings in vitro.

**Results:**

SS dose- and time-dependently induced liver injury in mice, indicated by increased serum AST, ALT and LDH levels. According to proteomic analysis, 487 differentially expressed proteins were identified in mice administrated with different dose of SS for different time periods. Altered proteins were enriched in pathways such as lipid metabolism, protein metabolism, macro molecular transportation, cytoskeleton structure and response to stress. SS enhanced CYP2E1 expression in a time and dose dependent manner, and induced oxidative stress both in vivo and in vitro.

**Conclusion:**

Our results identified hepatotoxicity and established dose-time course-liver toxicity relationship in mice model of SS administration and suggested potential mechanisms, including impaired lipid and protein metabolism and oxidative stress. The current study provides experimental evidence for clinical safe use of RB, and also new insights into understanding the mechanism by which SS and RB induced liver injury.

## Background

Radix Bupleuri (RB) is the dry root of *Bupleurum chinense* DC. (Apiaceae) and *Bupleurum scorzonerifolium* Willd. It represents one of the most successful herbal drugs in China and other Asian countries and has been widely used as a treatment for many diseases over the past 2000 years. It has effects on cold fever, chill and fever in turn, the feeling of oppression and illness in the chest and hypochondria [1, 2]. Furthermore, RB has been popularly used to treat many liver diseases such as chronic hepatic inflammation and viral hepatitis [3]. The widely prescribed Chinese herbal product, *Xiao-Chai-Hu-Tang*, a famous multi-herbal remedy containing RB, is renowned for its possible healing effects on chronic hepatitis B and its beneficial effects on preventing the development of hepatocellular carcinoma in patients with liver cirrhosis [4–6]. A study performed in Hong Kong has shown that 39% patients with chronic liver diseases prefer to use Chinese herbal products and 21% and 13% patients have taken Traditional Chinese Medicine (TCM) previously or are currently using TCM to improve their liver conditions, respectively [7]. According to the Chinese pharmacopoeia, the clinical safe dosage of RB prescriptions ranged from 3 g/day to 10 g/day, based on 70 kg body weight. However, based on accumulating evidence, RB probably contributes to hepatotoxicity, particularly overdose-induced acute liver injury and accumulation-related hepatotoxicity [8–11]. Patients using *Xiao-Chai-Hu-Tang* and *Long-Dan-Xie-Gan-Tang* or Chinese herbal products containing more than 19 g of RB might were recently shown to have an increased risks of liver injury [3]. Based on consecutive reports of the adverse hepatotoxic effects of RB, increasing concerns about its effectiveness and safety have been raised.

Saikosaponins (SS) are oleanane type triterpenoid saponins, and are the major bioactive compounds isolated from RB [12]. SS exhibits anti-inflammatory, anti-tumor, anti-viral, immunoregulatory and hepatoprotective effects [13]. Our previous study demonstrated that SS contributes to RB-induced chronic and acute hepatotoxic effects on rats and mice [14–17]. A statistically significant linear time- and dose-dependent trends for SS-induced liver toxicity were identified [14]. However, the molecular mechanisms underlying the hepatotoxicity of SS and its molecular targets are still unclear.

Proteomic technologies are large-scale research tools that provide abundant data regarding protein expression patterns, and are widely used to explore the molecular mechanisms of complex bioactive mixtures, including TCM. Classical 2DE has been commonly used for liver injury proteomics, but drawbacks have also been noted, such as low sensitivity, the extensive time required to complete procedure, and for the failure to detect low-abundance proteins [18, 19]. Recently, a new method, iTRAQ labelling coupled with LC–MS/MS, which is more sensitive, automatic, and multidimensional, has been applied to detect a large range of molecules (>20 kDa) and is more suitable for the study of pathogenic mechanisms and pathophysiology of diseases [20, 21].

In the current study, the liver toxicity of SS was first identified using a histopathologic evaluation and serum biochemistry assays. The iTRAQ proteomic technology was then employed to study the expression of SS-regulated proteins in the mouse liver. The identification of these differentially expressed proteins not only revealed time- and dose-related patterns of SS-induced hepatotoxicity but also candidate protein targets and signaling pathways, which provide novel insights into the underlying mechanism.

## Methods

### Preparation of SS from RB

In accordance with Chinese pharmacopoeia, and GMP standards, RB was purchased from Shandong Baiweitang (Jinan, Shandong), and authenticated by Professor Lin Hui-bin, Shandong Academy of TCM. The method used to prepare an alcohol extract of Bupleurum SS is described below: The samples were first extracted with 65% alcohol; the prepared extract was then recovered with alcohol and concentrated. Following purification on a D101 macroporous resin column, the 70% alcohol extract of the concentrated solution was collected. The crude drug content was 12.0 g/mL and the total SS content was 972.8 mg/mL. After the extract was air-dried under reduced pressure, the samples were diluted to the required concentration in a suspension with saline for animal expreiments, or phosphates buffered saline (PBS) for in vitro experiments.

### Phytochemical analysis of the extracts

Saikosaponins were prepared for High Performance Liquid Chromatography (HPLC) analysis by filtering through 0.45 μM membrane. Sakosaponin A (SSa) and Saikosaponin D (SSd) were separated on a Thermo Synecrosis C18 column (5 mm, 4.6 mm × 250 mm). SHIMADZU LC-20AT equipped with UV/VIS detector was used. The mobile phase consists of two solvents: Acetonitrile (A) and water (B). The following gradient programs were set: from 25% A to 90% A in 50 min and 90% A for 5 min. The detection wavelength was set to 210 nm.

### Animals and study design

Kunming mice weighing (20 ± 2) g of both sexes were purchased from the Experimental Animal Breeding and Research Center, Shandong University ([SCXK (Lu)20,090,001]). The mice were then housed in cages by gender under conditions of constant humidity (55 ± 5)%, temperature (22 ± 2) °C, a 12 h light/dark cycle and water ad libitum. All animal experiments were conducted in accordance with institutional guidelines and ethics.

For the time-toxicity study, 80 mice were divided into 7 groups including 0, 1, 2, 4, 8, 12, 24 and 48 h groups. The mice were intragastrically administered with saline (vehicle control) or SS at dosage of 21.650 g/kg of body weight. For dose-toxicity study, 40 mice were divided into 5 groups and administrated different doses of SS for 24 h, including saline (vehicle control), VL (4.675 g/kg of body weight), L (7.925 g/kg), M (12.957 g/kg), H (21.650 g/kg) and VH (36.075 g/kg) groups. At the end of the treatment, the mice were sacrificed and livers were collected. Protein concentrations were determined by BCA Protein Assay Kit (Beyotime Biotech, China). Blood was collected for biochemistry analysis. Serum levels of alanine aminotransferase (ALT), aspartate aminotransferase (AST), lactic acid dehydrogenase (LDH), total cholesterol and triglyceride (TG) were determined. All assay kits were purchased from Jiancheng Bioengineering Institute (China).

### iTRAQ labelling and 2D LC-MS/MS Analysis

The iTRAQ labelling was performed according to the manufacturer’s protocol (Applied Biosystem Inc., Foster city, CA). Briefly, 100 μg of proteins were prepared with iTRAQ™ dissolution buffer (ABI, Foster City, USA). After reduction and alkylation, protein solutions were digested overnight with sequencing-grade modified trypsin (Sigma Co. USA). The peptides were then labelled with iTRAQ regents. The samples were desalted with Sep-Pak Vac C18 cartridges (Waters, Milford, MA) and dried in a vacuum concentrator.

The mixture of iTRAQ labelling peptides was fractionated by strong cation exchange (SCX) chromatography on a 20 AD HPLC system (Shimadzu; Kyoto, Japan) using a Polysulfoethyl column (2.1 × 100 mm, 5 μm, 200 Å, The Nest Group, Southborough, MA). The peptide mixtures were reconstituted in Buffer A (10 mM KH_2_PO_4_ in 25% ACN (Fisher scientific, Fair Lawn, New Jersey)), loaded into the column and were separated at a flow rate of 200 μl/min for 60 min with a gradient of 0–80% Buffer B (Buffer A containing 350 mM KCl) in Buffer A. The absorbance at 214 nm and 280 nm was monitored and a total of 8 SCX fractions were collected. The fractions were vacuum dried and then resuspended in 50 μL of HPLC Buffer A (5% ACN, 0.1% formic acid (TEDIA, Fairfield, USA)), loaded across the ZORBAX 300SB-C18 reversed-phase column (5 μm, 300 Å, 0.1 × 150 mm; Microm, Auburn, CA) and analyzed on a Triple Tof 5600 System (Applied Biosystem, USA) coupled with a 20 AD HPLC system (Shimadzu; Kyoto, Japan). The flow rate for elution was 0.3 μL/min using a 5%–35% gradient of HPLC Buffer B (95% ACN, 0.1% formic acid) for 120 min. The survey scans were obtained with m/z ranges of 400–1500, for MS with up to four precursors were selected from the m/z 100–2000 region for MS/MS.

### Proteomic data analysis and bioinformatics

The MS data were extracted and searched against the Swiss Prot database (20,090,303 released) using the ProteinPilot software (Applied Biosystem, USA) to identify and quantify the peptides and proteins. The Paragon Algorithm and the Pro Group trypsin lgorithm (Applied Biosystem, USA) were sequentially applied to determine the final identification of the proteins. Autobias was assessed using protein pilot to eliminate some differences caused by the experimental process. An unused ProtScore >1.3and more than one peptide above the 95% confidence interval were set as threshold for protein identification. False Discover Rate (FDR) for protein detection was calculated as FDR = (2 × reverse)/ (forward + reverse). The global FDR of the combined data was 1%. The biological processes were annotated by Gene Ontology (GO) database and KEGG database and manually slimed. Toxigenomics analysis was conducted using Comparative Toxigenomics Database. Clustering and enrichment analyses were performed as described previously. [22].

### Redox status assessment

GSH and GSSG assay kit (Beyotime Biotech, China), reactive oxygen species (ROS) assay kit, Maleic Dialdehyde (MDA) assay kit and iNOS assay kit (Jiancheng Biotech, China) were used to determine the oxidative stress level in liver or cell. All the results were normalized to protein concentrations for animal studies or normalized to cell numbers for in vitro experiments.

### Western Blot analysis

Total cell lysate from liver tissue were prepared using RIPA buffer. The protein concentrations were determined using Bio-Rad protein assay kit. The protein expression levels of CYP2E1 and GAPDH in liver samples were determined by Western Blot using specific primary antibody (Santa Cruz, CA, USA), as described previously [23].

### Cell culture and cell experiment

HepG2 cell line was purchased from ATCC and were cultured in Dulbecco’s modified Eagle’s medium (DMEM) medium in supplement with 10% fetal bovine serum (FBS), penicillin G (100 U/mL), streptomycin (100 μg/mL). All cell culture supplies were obtained from Gibco (Waltham, MA). HepG2 cells were treated with PBS (vehicle control) or different concentration of SS (25 μg/mL, 50 μg/mL, 100 μg/mL, 200 μg/mL and 400 μg/mL) for 12 h or 24 h. At the end of treatment, images of cells were taken. Cell viabilities were determined using Cell Counting Kit-8 (Dojindo, D.C. USA), according to manufacturer’s instruction. Intracellular ROS, GSH levels and iNOS activity were determined, as described above (Method 2.6 Redox status assessment).

### Statistical analysis

All the data are represented as Mean ± SEM. One-way ANOVA and Dunnett’s t-test were employed to analyze the differences between sets of data. A value of *P* < 0.05 was considered statistically significant.

## Results

### Phytochemical analysis

**Fig. 1:**
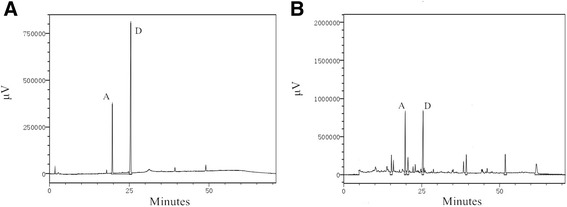
HPLC chromatograms. **a** Standard mix and **b** Alcohol elution of SS. SSa and SSd were indicated by “A” and “D”, respectively

HPLC was employed to analyze the typical chromatograms of SS extracts from RB. SSa and SSd Standards were used to identify the components. According to the chromatogram, the major SS were identified from a comparison with the retention times of external standards (SSa: 19.696 min, SSd: 25.473 min) and presented peaks at 19.722 min for SSa, and 25.446 min for SSd (Fig. 1). SSa and SSd constituted 20.499% and 26.679% of SS sample respectively, according to the calculation of peak area. More information is required for the identification of other phytochemicals, including SSb1, SSb2 and SSc.

### SS induces acute liver injury in mice

**Fig. 2:**
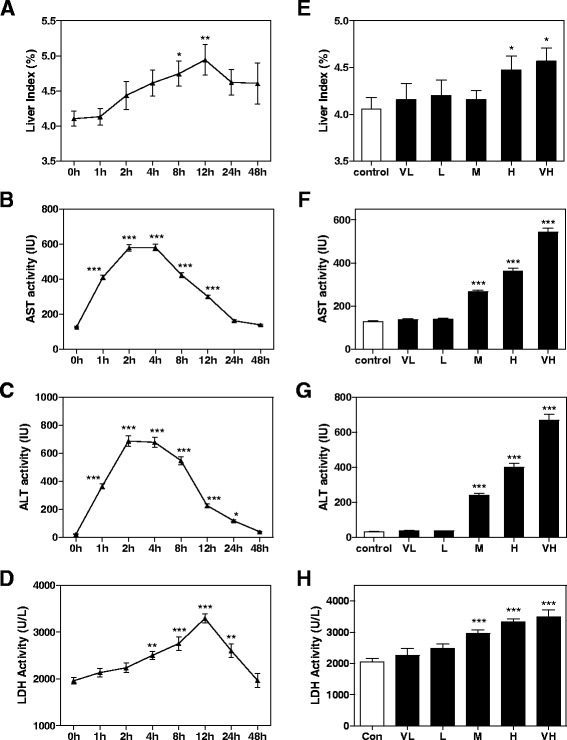
SS-induced liver toxicity. **a** and **e** liver index, Serum level of **b** and **f** AST, **c** and **g** ALT and **d** and **h** LDH after different time and dose of SS administration. All the data are presented as mean ± S.E. (*n* = 8), * p < 0.05, **p < 0.01, ***p < 0.001, compared to time 0 h or vehicle control group, respectively

After SS administration, mice were sacrificed as described in methods. As shown in Fig. 2a, the liver index (liver weight/body weight) was significantly increased beginning at 8 h after SS administration. Due to the relative short period of SS administration, no appreciable changes were observed during the histopathological examination using hematoxylin and eosin staining (data not shown), with the exception of occasional focal hepatocyte necrosis and inflammatory cell infiltration in the H group. As shown in Fig. 2b and c, serum AST and ALT levels were significantly elevated as early as 1 h after the mice were treated with SS, reached peak at approximate 4 h, and gradually recovered from 24 h to 48 h. Serum LDH activity level, which indicated an impairment of hepatocyte membrane integrity, was also increased at 4 h, and reached peak at 12 h (Fig. 2d). As shown in Fig. 2e-h, we further demonstrated that SS dose dependently induced elevation of these liver injury makers.

### Analysis of SS-induced differentially expressed proteins

**Fig. 3:**
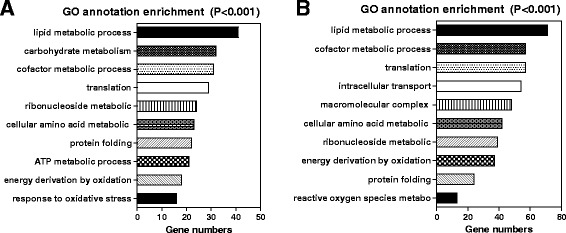
GO analysis for SS-induced differentiated expressed proteins. GO annotation enriched biological pathways for (**a**) time-toxicity study and **b** dose-toxicity study

We then used an iTRAQ reagent-based quantitative proteome analysis as a global approach to investigate potential proteins and pathways associated with SS-induced liver injury in mice. 1288 proteins were quantified and further used for bioinformatics analysis. In the time-dependent toxicity study, 332 proteins were identified as significantly differentially expressed (fold change > 2, *p* < 0.05), including 149 up-regulated proteins, and 183 down-regulated proteins. According to the GO annotation, the enriched biological pathways were mainly involved in lipid metabolism, carbohydrate metabolism, cofactor metabolic process, protein translation and metabolism, energy homeostasis and cellular response to stress (Fig.3a). In the dose-toxicity study, 654 proteins were identified significantly differentially expressed (fold change > 2, *p* < 0.05). Dose-toxicity study (Fig. 3b) further suggested that SS induced liver toxicity through multiple mechanisms in a time- and dose-related manner.

### Time course analysis of SS-regulated biological pathways

**Fig. 4:**
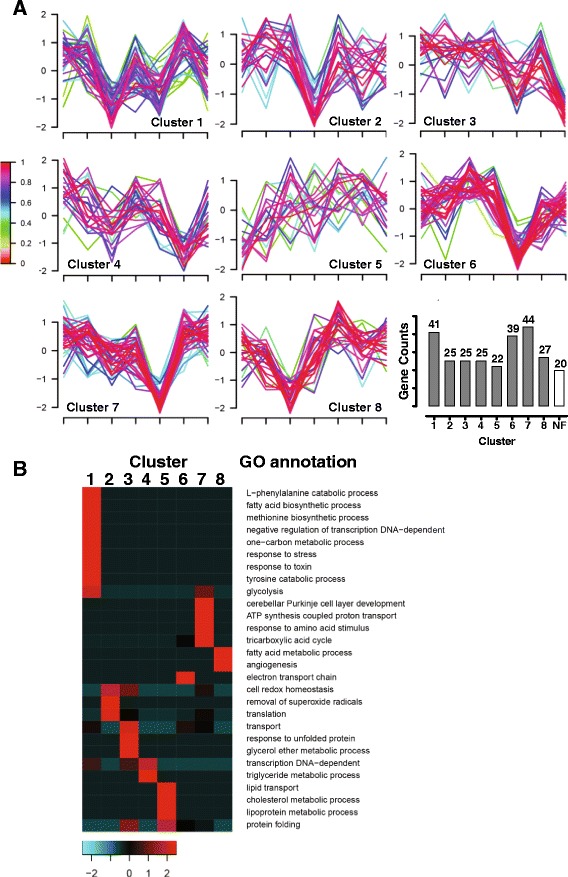
Cluster analysis of SS-regulated proteins in time-toxicity study. **a** Cluster analysis and profiles. Legend (left) indicates fitness to cluster center. Numbers of protein counts in each cluster were plotted with cluster number (NF indicates non-fit proteins). **b** Enrichment of GO biological pathways to cluster profiles

In order to identify the causes and consequences of SS-induced liver toxicity, we clustered all differentially expressed proteins observed in the time-toxicity study based on their fold changes over time. 292 proteins out of 302 proteins fit into 8 patterns (Fig. 4a). We then conducted enrichment analysis of GO biological pathways based on the different clustering patterns. According to the enrichment results (Fig. 4b), proteins involved in lipid transportation, lipoprotein metabolic process, and the fatty acid metabolic process were all rapidly up- or down-regulated from 1 h to 2 h after SS-administration (Cluster 5 and 8). Proteins involved in the response to stress or toxin, amino acid metabolism and carbohydrate metabolism were significantly regulated at 2 h or 6 h after SS-administration and recovered after 24 h or 48 h (Cluster 1 and 2). 12 h after SS administration, energy homeostasis, including ATP synthesis, the tricarboxylic acid cycle and electron transport chain, was significantly disrupted. In addition, protein translation and protein folding processes were also regulated by SS from 2 h to 6 h after administration. Intracellular transport, macromolecular complex metabolic processes were regulated 12 h after SS exposure and persisted until 24 h to 48 h. Based on this evidence, dysregulation of lipid transportation and metabolism occurred prior to, and is a plausible cause of the disruption of other biological pathway, including response to stress, energy homeostasis and protein homeostasis.

### Effects of SS on lipid transport and metabolism

**Fig. 5:**
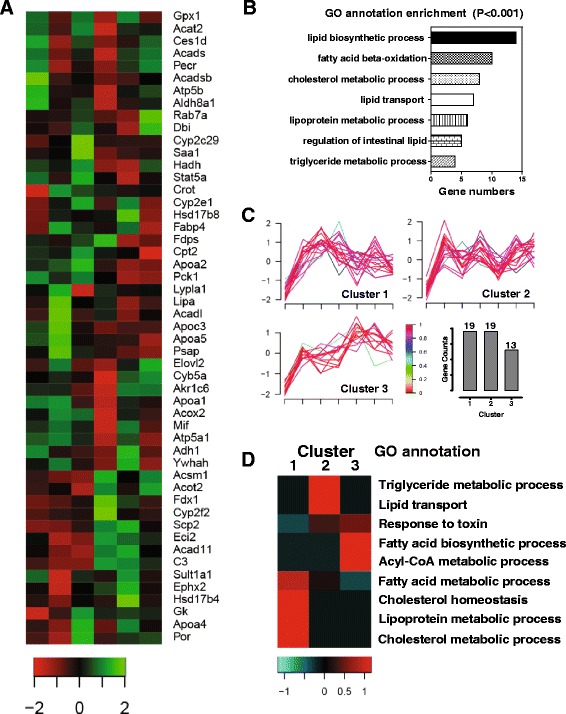
Cluster analysis of SS-regulated lipid metabolism related proteins. **a** The list of SS-regulated proteins involved in lipid metabolism. Legend indicates fold changes (Log). **b** GO annotation enriched biological pathways based on selected proteins. **c** Cluster analysis and profiles for selected proteins. **d** Enrichment of GO biological pathways to cluster profiles

Because dysregulation of lipid metabolism is the most common features of several liver diseases, we identified 51 proteins enriched in the lipid metabolic process pathway (Fig. 5a). According to the cluster analysis, the expression of genes related to lipoprotein metabolism, cholesterol homeostasis and fatty acid metabolic processes were up-regulated rapidly and then down-regulated (Cluster 1), whereas, genes related fatty acid biosynthetic and acyl-CoA metabolism were consistently increased (Cluster 2 and 3) (Fig. 5b-d). As summarized in Table 1, apolipoprotein A (Apo A) - I, II, IV, and V were all significantly upregulated and reached peak earlier than 8 h. ApoA- IV increased 3 folds 2 h after SS administration, which was the most sensitive and intensive, and persisted more than 48 h. All of these lipoproteins have been shown to participate in lipid transport from other organs to the liver by forming High-density lipoprotein or chylomicron particles, and are also involved in inducing of lipoprotein degradation and lipid metabolism. Additionally, proteins involved in triglyceride (TG) and cholesteryl esters hydrolysis, including Lipase A, were rapidly upregulated at 2 h after SS administration, which suggested that SS increased TG clearance and fatty acid production in hepatocytes and induced lipotoxicity.

**Table 1:** Effects of SS on expression of lipid metabolism related proteins

Protein name	protein ID	fold change	time(h)	protein description
Effect of SS on expression of Lipid transportation related proteins				
Apoa4	P06728	3.28	8	Apolipoprotein A-IV
Apoa2	P09813	2.22	8	Apolipoprotein A-II
Apoa1	Q00623	1.47	4	Apolipoprotein A-I
Apoa5	Q8C7G5	1.06	2	Apolipoprotein A-V
Saa1	P05366	-1.85	24	Serum amyloid A-1 protein
Effect of SS on expression of Lipid metabolism related proteins				
Lipa	Q9Z0M5	2.12	2	Lipase A
Fabp3	P11404	2.86	24	Fatty acid-binding protein
Scp2	P32020	1.33	24	Non-specific lipid-transfer protein
Fdx1	P46656	1.89	24	Adrenodoxin, mitochondrial
Acad11	Q80XL6	1.69	24	Acyl-CoA dehydrogenase family member 11
Acot2	Q9QYR9	1.20	48	Acyl-coenzyme A thioesterase 2, mitochondrial
Fdps	Q920E5	-1.55	24	Farnesyl pyrophosphate synthase
Sec14l2	Q99J08	-1.86	24	SEC14-like protein 2
Acsm1	Q91VA0	-1.69	8	Acyl-coenzyme A synthetase ACSM1
Dbi	P31786	-1.29	4	Acyl-CoA-binding protein
Acadl	P51174	-1.33	24	Long-chain specific acyl-CoA dehydrogenase

Fold change: peak fold change of certain protein expression

Time(h): time point after SS administration when reach peak fold change

All fold changes are relative to Control group (time point 0 h)

**Fig. 6:**
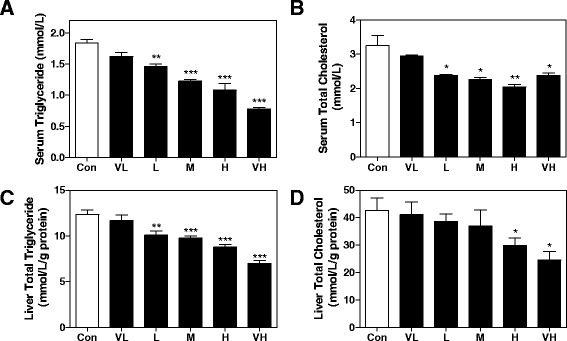
Effect of SS on lipid contents in serum and liver. **a** TG and **b** total cholesterol in serum, and **c** TG, **d** total cholesterol level in liver. All results are presented as mean ± S.E. (*n* = 8), * p < 0.05, **p < 0.01, ***p < 0.001, compared to vehicle control group

On the other hand, the up-regulation of several intracellular lipid transporters and components involved in fatty acid oxidation, such as fatty acid-binding protein, non-specific lipid-transfer protein, Acyl-CoA dehydrogenase family member 11 and Acyl-coenzyme A thioesterase 2, Cytochrome P 2E1 (CYP2E1), accompanied with down-regulation of other key enzymes or mediators of lipid metabolism, like Farnesyl pyrophosphate synthase, SEC14-like protein 2, Acyl-coenzyme A synthetase ACSM1, Acyl-CoA-binding protein and Long-chain specific acyl-CoA dehydrogenase, indicated dysregulation of hepatic lipid metabolism. Most of these proteins responded to SS after 12 h, and reached peak at approximately 24 h. As shown in Fig. 6, we observed dose-dependent decrease of serum and liver TG and cholesterol level 24 h after SS administration, indicating that excessive lipid oxidation neutralized increased lipid import in the liver.

### Effects of SS on the induction of oxidative stress

**Fig. 7:**
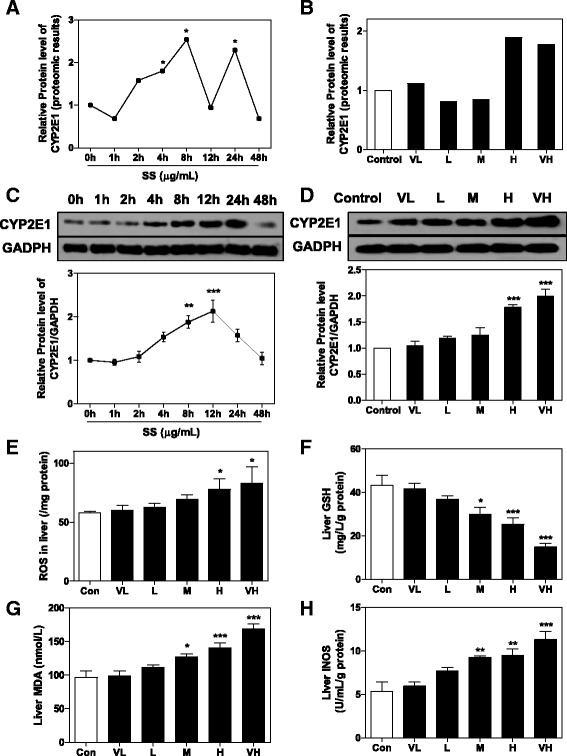
SS-induced oxidative stress in mice liver. Proteomic results for CYP2E1 expression in **a** time-toxicity study and **b** dose-toxicity study. * *p* value < 0.05. Proteins levels of CYP2E1 in **c** time-toxicity study and **d** dose-toxicity study were determined by western blot. Representative images of immunoblots are shown and analyzed using GAPDH as loading control. Effects of different dose of SS administration on **e** ROS, **f** GSH, **g** MDA and **h** iNOS level in liver. All results are presented as mean ± S.E. (*n* = 8),* p < 0.05, **p < 0.01, ***p < 0.001, compared to vehicle control group

Emerging evidence supported that oxidative stress is one of the most important mechanisms that directly induces damage during drug-induced liver injury. Proteins involved in the removal of superoxide radicals and the response to reactive oxygen species (ROS) were first upregulated at 1 h to 2 h and then significantly downregulated at 6 h after SS administration (Fig. 4a, b). Sod1, Gpx1, Tgm2, Gsta3, Gstp1 and Dhe3 were all downregulated more than 2 fold 6 h after SS administration **(**Table 2 **)**. CYP2E1 is particularly susceptible to ROS production, and links dysregulated lipid metabolism to oxidative stress. Protein levels of CYP2E1 were significantly increased 2 h after SS administration, reached peak at 8 h and persisted until 24 h (Fig. 7a, c). The induction of CYP2E1 expression was also dose dependent (Fig. 7b, d). In support of these findings, intra-hepatocytes ROS levels were significantly increased 12 h after SS administration in a dose-dependent manner, and the Glutathione (GSH) level in liver was significantly decreased (Fig. 7e, f). Oxidative stress was further confirmed by dose-dependent elevation of the Malondialdehyde (MDA) level, and increased iNOS expression (Fig. 7g, h).

**Table 2:** Effect of SS on expression of proteins related to response to oxidative stress

Protein name	protein ID	High	low	protein description
Ggt1	Q60928	1.30	0.74	Gamma-glutamyltranspeptidase 1
Sod1	P08228	1.25	0.58	Superoxide dismutase [Cu-Zn]
Gpx1	P11352	1.57	0.17	Glutathione peroxidase 1
Cycs	P62897	1.38	1.00	Cytochrome c, somatic
Glo1	Q9CPU0	1.33	0.60	Lactoylglutathione lyase
Rgn	Q64374	-1.13	−0.65	Regucalcin
Bag5	Q8CI32	-1.47	−0.35	BAG family molecular chaperone regulator 5

High/Low: indicate fold changes of certain proteins after high dose or low dose of SS administration

All fold changes are relative to Control group

**Fig. 8:**
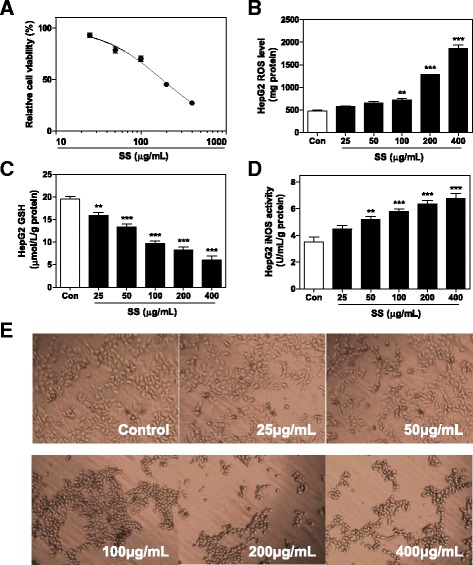
Effects of SS on HepG2 cells. **a** Effects of SS on cell viability of HepG2 cells after 24 h treatment. Effects of 12 h treatment of SS on **b** ROS, **c** GSH and **d** iNOS level in HepG2 cells. **e** Effects of SS on morphology changes of HepG2 cells after 12 h treatment. All results are presented as mean ± S.E. (*n* = 3), **p < 0.01, ***p < 0.001, compared to vehicle control group

HepG2 cells were treated with different dose of SS (from 25 μg/mL to 400 μg/mL) to further confirm the oxidative stress inducing effects of SS. As expected, SS significantly induced cell death of HepG2 24 h after administration, with an IC50 less then 200 μg/mL (Fig. 8a). Intracellular ROS levels were dose-dependently increased 12 h after treatment, accompanied with depleted GSH level and increased iNOS activity (Fig. 8b-d). All these changes suggested that SS-induced excessive but dysregulated lipid metabolisms leaded to increased ROS production and following oxidative stress.

### Effects of SS on protein translation and degradation

Enriched GO biological pathways analysis also suggested altered protein translation and metabolism in the SS-treated mouse liver. As summarized in Table 3, proteins required for mRNA maturation and small nuclear ribonucleoprotein biogenesis, were either up-regulated or down-regulated within a relatively short period after SS exposure. In addition, several ribosome components were significantly downregulated beginning at 4 h after SS administration, reached peak at 8 h that persisted for over 24 h. The disruption of the ribosome will eventually leads to a global inhibition of protein translation and contributes to the loss of cellular functions. Additionally, correct protein folding is essential for protein maturation and biological function. However, our proteomic analysis indicated that several proteins involved in assisting protein folding in the endoplasmic reticulum were down-regulated. Furthermore, the levels of several proteasome components were increased, indicating that the protein degradation mechanism may have been up-regulated. All these confusion results demonstrated that the protein homeostasis and regular protein translation-folding-degradation processes were disrupted by SS and may have contributed to the failure of the hepatocyte repair mechanism and subsequent apoptosis.

**Table 3:** Effects of SS on expression of protein metabolism related proteins

Protein name	protein ID	fold change	time(h)	protein description
Effect of SS on expression of translation related proteins				
Sfrs5	O35326	1.24	4	Splicing factor, arginine/serine-rich 5
Sfrs7	Q8BL97	1.49	2	Splicing factor, arginine/serine-rich 7
Ptbp1	P17225	1.59	2	Polypyrimidine tract-binding protein 1
Ruxe	P62305	-1.38	24	Small nuclear ribonucleoprotein E
Smd2	P62317	-1.17	2	Small nuclear ribonucleoprotein Sm D2
Npm1	Q61937	-1.09	8	Nucleophosmin
Rps16	P14131	-2.29	8	40S ribosomal protein S16
Rpsa	P14206	-1.41	8	40S ribosomal protein SA
Rps14	P62264	-1.08	8	40S ribosomal protein S14
Rps24	P62849	-1.09	8	40S ribosomal protein S24
Rps28	P62858	-1.32	24	40S ribosomal protein S28
Rps19	Q9CZX8	-1.40	24	40S ribosomal protein S19
Effect of SS on expression of post-translation modification related proteins				
Pmpcb	Q9CXT8	3.51	8	Mitochondrial-processing peptidase subunit beta
Hspa2	P17156	1.50	8	Heat shock-related 70 kDa protein 2
Hsp90ab1	P11499	-1.28	8	Heat shock protein HSP 90-beta
P4hb	P09103	-1.33	4	Protein disulfide-isomerase
Pdia3	P27773	-1.27	24	Protein disulfide-isomerase A3
Pdia6	Q922R8	-2.38	8	Protein disulfide-isomerase A6
Canx	P35564	-1.38	8	Calnexin
Erp44	Q9D1Q6	-1.91	8	Endoplasmic reticulum resident protein 44
Psmd14	O35593	1.37	8	26S proteasome non-ATPase regulatory subunit 14
Pmsa5	Q9Z2U1	1.53	4	Proteasome subunit alpha type-5
Psmb9	P28076	1.23	24	Proteasome subunit beta type-9

Fold change: peak fold change of certain protein expression

Time(h): time point after SS administration when reach peak fold change

All fold changes are relative to Control group (time point 0 h)

### Effects of SS on cellular organization and intracellular transport-related proteins

The cellular organization and intracellular transport systems are not only critical for macromolecular uptake and metabolism, but are also essential for other functions such as bile secretion and uptake in hepatocytes. Our proteomic results demonstrated that several important proteins related to cytoskeleton stabilization were dose dependently decreased 8 h after SS administration. Consistent with these findings, significant morphological changes of HepG2 cells, from a polygon to a shrinking circle, were observed 12 h after SS treatment, prior to cell death (Fig. 8e). In addition, several intracellular transport-related proteins were also significantly down-regulated as well **(**Table 4 **)**. The disruption of cytoskeleton stability and disturbance of intracellular transport directly lead to hepatocyte dysfunction and following liver injury.

**Table 4:** Effect of SS on expression of cytoskeleton organization and intra-cellular transportation related proteins

Protein name	protein ID	High	low	protein description
Gsna	P13020	-2.44	-0.44	Gelsolin
Flna	Q8BTM8	-1.35	-0.21	Filamin-A
Lpin1	Q91ZP3	-1.46	-1.40	Phosphatidate phosphatase LPIN1
Racgap	Q9WVM1	-2.35	-1.71	Rac GTPase-activating protein 1
Chp1	P61022	-2.86	-1.42	Calcineurin B homologous protein 1
Rab10	P61027	-4.01	-1.06	Ras-related protein Rab-10
Rab-7a	P51150	-1.77	-0.54	Ras-related protein Rab-7a
Rab-5c	P35278	-2.59	-0.70	Ras-related protein Rab-5C
Nras	P08556	-2.52	-0.61	GTPase Nras
Vps4b	P46467	-2.25	-0.19	Vacuolar protein sorting-associating protein 4B
Cit	P49025	-1.55	-0.73	Citron Rho-interacting kinase

High/Low: indicate fold changes of certain proteins after high dose or low dose of SS administration

All fold changes are relative to Control group

## Discussion

As demonstrated in our previous studies, SS are primary ingredients responsible for RB-induced hepatic adverse effects [15, 16]. In the current study, SS induced time- and dose-dependent acute liver injury in mice. Dysfunction of lipid metabolism and dysregulation of lipid homeostasis were critical causes, as well as consequences of liver injury. In addition to the up-regulation of Apolipoproteins, the transport of TG and cholesterol from other organs to liver for oxidation or secretion was significantly increased in a short time period after SS administration. The increased levels of critical enzymes involved in TG and cholesterol hydrolysis such as Lipase A, further accelerated lipid clearance from the circulation and liver. Under normal conditions, the cleavage of TG and cholesterol significantly reduced risk of atherosclerosis, alleviated insulin resistance and maintained liver and body lipid homeostasis, suggesting potential pharmacological effects of SS [24, 25]. However, the regulation of fatty acid metabolism in the liver was paradoxically dysregulated after SS administration. Proteins involved in fatty acid uptake and β-oxidation, such as Acot2 and Acad11, were up-regulated, whereas other proteins, such as Acad1, were significantly down regulated [26]. When the SS-induced excess lipids are imported into the liver, this disordered expression will subsequently disturb fatty acid metabolism in hepatocytes and induce lipotoxicity.

Emerging evidence supported that dysregulation of lipid metabolism, intracellular accumulation of fatty acids and impairments of fatty acid β-oxidation presumably stimulated ROS production by promoting electron overflow in the mitochondrial respiration chain [27, 28]. Excessive ROS levels overwhelmed the anti-oxidation mechanism, and will then damage hepatocytes [29]. Furthermore, the significant induction of CYP2E1 expression by SS was an important source of ROS production by enhanced omega fatty acid oxidation [30]. As a consequence of unresolved oxidative stress, significant lipid peroxidation, which has been characterized as an important cause and marker of drug-induced liver injury due to its critical role in membrane integrity impairment, was also observed by monitoring liver MDA level in our study. Oxidative stress significantly induced iNOS expression, which further contributed to liver dysfunction and damage by induction of chronic inflammation and endothelial disruption. Mitochondrial membrane potency was also significantly disrupted as a consequence of excessive oxidative stress (Data not shown). On the other hand, the levels of some proteins that protect cells against mitochondrial damage or apoptosis, including Bag5 and Rgn, were remarkably down-regulated [31, 32]. These results provided important evidence suggesting that ROS production following oxidative stress and related damage serves as an important mechanism in SS-induced liver injury.

The accumulation of fatty acids, particularly long-chain and saturated fatty acids, has been suggested to be involved in inducing endoplasmic reticulum stress and disrupting lipid metabolism in liver diseases [33]. Fatty acids-induced oxidative stress, disturbances of calcium homeostasis and altered membrane lipid saturation were considered to be three main mechanisms [34, 35]. Apoptosis driven by CHOP and JNK activation will be triggered once cells failed to recover from endoplasmic reticulum stress [36]. Furthermore, our proteomics results suggested that the mechanism by which SS disrupted protein expression was more complicated and severe. Global protein dysregulation will then induce cytoskeletal disorganization, dysfunctional intracellular transport and also impaired membrane organization [37]. Dysregulation of protein metabolism not only disrupted normal liver function but also interfered with the hepatoprotective and recover mechanism of liver against stress, subsequently leaded to SS-induced hepatocytes apoptosis or necrosis, consistent with clinical and pathological findings [9].

Interestingly, according to our dose-toxicity study, significant acute liver injury only occurred when the animals were administered a dose greater than 12.957 mg/kg, which is approximately 8 folds higher than the safety daily dose used clinically. This finding highlighted the risks of adverse effects following an acute overdose of RB-containing prescriptions, and provided experimental evidence of a quantified dose-toxicity relationship, which will promote the safe clinical use of RB-containing products. Further studies are still required to elucidate the plausible mechanism underlying long-term consumption of RB-induced toxicity. Critical biological pathways identified in this acute liver injury model are also likely involved in the chronic hepatotoxicity of SS. Furthermore, it was noteworthy that in L group, although no significant liver injury was observed, several biological pathways, including lipid transportation and metabolism, were still significantly regulated by SS administration. In support of this finding, several studies demonstrated that relatively low dose of SS alleviated chronic liver diseases, including fatty liver, fibrosis, cancer or chemical-induced liver injury [38]. These findings suggested that SS-induced bioactive effects, either pharmacological or toxicological, were dose sensitive. In contrast to maintaining hepatic metabolism homeostasis and hepatoprotective effects of SS at a pharmacological dose, SS overdose induced excess disturbances of several vital biological functions and resulted in liver injury. In addition to critical control of dose during clinical practice, compatibility art of TCM provides another common strategy to attenuated potential toxicity of RB. Hepatic protective herbs, including licorice, were widely used as herb pairs with RB in famous Chinese herbal formulas, *Chai-Hu-Shu-Gan-San*, *Long-Dan-Xie-Gan-Tang* and *Xiao-Chai-Hu-Tang*, for the treatment of hepatitis, cold and fever [39–42].

## Conclusion

In conclusion, SS induced severe dysregulation of lipid metabolism and protein expression, which further presumably induced excess ROS generation and hepatocyte apoptosis. Several plausible mechanisms, including lipid metabolism pathways, oxidative stress, mitochondrial damage and dysregulation of global protein metabolism are subjects for further studies. In addition to establishing dose- time course-liver toxicity relationship in a mouse model, the current study provides experimental evidence for the safe clinical use of RB-containing remedies, and new insights into understanding the mechanisms by which SS and RB induce hepatotoxicity.

## Abbreviations

ApoA: Apoliprotein A
CYP2E1: Cytochrome P 2E1
GSH: Glutathione
MDA: Malondialdehyde
RB: Radix Bupleuri
ROS: Reactive oxygen species
SS: Saikosaponins
TCM: Traditional Chinese Medicine
TG: Triglyceride
